# Multiple substrate recognition by yeast diadenosine and diphosphoinositol polyphosphate phosphohydrolase through phosphate clamping

**DOI:** 10.1126/sciadv.abf6744

**Published:** 2021-04-23

**Authors:** María Ángeles Márquez-Moñino, Raquel Ortega-García, Megan L. Shipton, Elsa Franco-Echevarría, Andrew M. Riley, Julia Sanz-Aparicio, Barry V. L. Potter, Beatriz González

**Affiliations:** 1 Department of Crystallography and Structural Biology, Institute of Physical-Chemistry Rocasolano, CSIC, Serrano 119, 28006 Madrid, Spain.; 2 Drug Discovery and Medicinal Chemistry, Department of Pharmacology, University of Oxford Mansfield Road, Oxford OX1 3QT, UK.

## Abstract

The yeast diadenosine and diphosphoinositol polyphosphate phosphohydrolase DDP1 is a Nudix enzyme with pyrophosphatase activity on diphosphoinositides, dinucleotides, and polyphosphates. These substrates bind to diverse protein targets and participate in signaling and metabolism, being essential for energy and phosphate homeostasis, ATPase pump regulation, or protein phosphorylation. An exhaustive structural study of DDP1 in complex with multiple ligands related to its three diverse substrate classes is reported. This allowed full characterization of the DDP1 active site depicting the molecular basis for endowing multisubstrate abilities to a Nudix enzyme, driven by phosphate anchoring following a defined path. This study, combined with multiple enzyme variants, reveals the different substrate binding modes, preferences, and selection. Our findings expand current knowledge on this important structural superfamily with implications extending beyond inositide research. This work represents a valuable tool for inhibitor/substrate design for *Sc*DDP1 and orthologs as potential targets to address fungal infections and other health concerns.

## Teaser

A NUDIX enzyme joins inositide and polyphosphate metabolisms through multisubstrate binding ability.

## INTRODUCTION

Inositol polyphosphates (InsPs) are small molecules, primarily derived from *myo*-inositol, the phosphorylation patterns of which generate second messengers, essential metabolites, or cofactors involved in a variety of cell processes (*1*). A subset of the InsPs is formed by the inositol pyrophosphates (PP-InsPs), essential for vesicular remodeling, growth, phosphate, and energy homeostasis (*2*–*4*) and, particularly in yeast, critical for pseudohyphal growth (*5*). In humans, they are necessary for correct cell physiology and are involved in diseases leading to diabetes and cancer (*6*–*8*). From yeast to mammals, PP-InsPs with seven phosphates (diphosphoinositol pentakisphosphate, PP-InsP_5_ or InsP_7_) and eight phosphates (bis-diphosphoinositol tetrakisphosphate, [PP]_2_-1,5-InsP_4_ or InsP_8_) have been identified, as well as two different isomers of InsP_7_ [1-InsP_7_ (or 1-PP-InsP_5_) and 5-InsP_7_ (or 5-PP-InsP_5_)] (*9*). A very recently published work shows that 5-InsP_7_ is an endogenous negative regulator of Na^+^/K^+^–adenosine triphosphatase (ATPase) α1 (*10*). Regulation of these metabolites is achieved by the action of kinases and phosphatases that balance correct levels in cells. Two different families of kinases are responsible for their synthesis from inositol hexakisphosphate (InsP_6_), the InsP_6_ kinases and the diphosphoinositol-pentakisphosphate kinases (PP-InsP_5_Ks; named as Vip1 in *Saccharomyces cerevisiae* or Asp1 in *Schizosaccharomyces pombe*). However, three different families of phosphatases that are able to hydrolyze PP-InsPs at least in vitro have been identified, belonging in terms of their fold to the Nudix hydrolase [human diphosphoinositol polyphosphate phosphohydrolases (DIPPs)], tyrosine phosphatase (*Sc*Siw14, essential for prion propagation), or histidine acid phosphatase (Vip1 enzymes) families. Regarding the latter, Vip1 and Asp1 yeast kinases are bifunctional enzymes containing tethered kinase and phosphatase domains.

In this work, we focus on the structural and functional features of diadenosine and diphosphoinositol polyphosphate phosphohydrolase (DDP1), a phosphatase that belongs to the Nudix hydrolase fold. DDP1 is a *S. cerevisiae* enzyme (also *Sc*DDP1) that is able to hydrolyze several kinds of substrates in vitro. As mentioned, DDP1 displays PP-InsP phosphatase activity (*11*, *12*). It hydrolyzes 1-InsP_7_, 5-InsP_7_, and InsP_8_, although it hydrolyzes 1-InsP_7_ faster than InsP_8_ and 5-InsP_7_ (*13*). However, *Sc*DDP1 is not exclusively a PP-InsP phosphatase, since it also hydrolyzes polyphosphates (polyPn) (*11*, *14*) and diadenosine polyphosphates (Ap*_n_*A as Ap_5_A or Ap_6_A) (*11*, *15*). *Sc*DDP1 participation in cytoplasmic polyPn cleavage has been already proven (*16*). This has an important implication since polyPs are related to stress adaptation and metal toxicity resistance in some fungal pathogens and in the cell cycle of *S. cerevisiae* by providing phosphate to deoxynucleotide triphosphates (dNTPs) synthesis for S phase (*17*). In humans, polyPs have health implications since they are relevant for platelet aggregation, inflammation, the immune system, and cancer (*18*–*20*). Similarly, Ap*_n_*As participate in cell proliferation, differentiation, and apoptosis equilibria, mediating extracellular stimuli (*21*). In higher organisms, these compounds can also act as neurotransmitters (*22*) or vasomodulators (*23*).

All these catalytic activities position *Sc*DDP1 at a key point for phosphate homeostasis through polyPs and InsP_7_ degradation (*24*). Previous data have shown that both metabolisms (polyPs and PP-InsPs) are highly correlated (*11*). A hot topic over the past years is that PP-InsPs may act as Pi sensors in the phosphate-responsive signaling (PHO) pathway through binding to SPX domains (from SYG1/Pho81/XPR1 proteins) (*25*). Thus, InsP_7_ in *S. cerevisiae* is necessary to activate the vacuolar transporter chaperone complex responsible for polyP synthesis and storage in yeast vacuoles and to control PHO transcription genes by activating the Pho81 cyclin-dependent kinase inhibitor (*17*, *26*). It has been shown that PP-InsPs can act as a glue for protein-protein complexes involved in PHO pathway (*25*). Certainly, the relationship of DDP1 with phosphorus homeostasis makes this enzyme a potential target for fungal infections, the virulence of which may depend on phosphorus as an essential nutrient (*17*). In parallel, *Sc*DDP1 may also remove toxic dinucleoside polyphosphates synthesized during sporulation (*27*).

*Sc*DDP1 presents orthologs in humans (the DIPP family) and in *S. pombe* (Aps1). Five orthologs of *Sc*DDP1 have been reported in humans (*28*): *Hs*DIPP1, *Hs*DIPP2, *Hs*DIPP3α, *Hs*DIPP3β, and *Hs*DIPP3γ. Up to now, the structures of several *Hs*DIPP isoforms are available, but only the *Hs*DIPP1 isoform has been published (*28*, *29*). These enzymes belong to the Nudix hydrolase superfamily, also known as the MutT/Nudix family. Briefly, the Nudix hydrolases are enzymes found in all organisms from all kingdoms and in viruses, having β-grasp domain architecture. They present diverse biological roles, being involved in cellular metabolism and homeostasis as well as in mRNA processing. This fold does not only serve exclusively the pyrophosphohydrolases but is also found in adenine/guanine mismatch-specific adenine glycosylases, isopentenyl diphosphate isomerases, and proteins with nonenzymatic activities such as protein interactions and transcriptional regulation (*30*). Most Nudix enzymes contain a 23–amino acid motif named the MutT/Nudix box, which, in pyrophosphatases, is GX_5_EX_7_REUXEEXGU, where U is a bulky aliphatic residue (L, I, or V) and X is any amino acid. This displays a loop-helix-loop structure that binds one or more metals involved in diphosphate moiety orientation and catalysis. Therefore, it happens that the Nudix homology domain is an effective scaffold for many catalytic activities. DDP1 shares sequence homology in a limited region with other pyrophosphohydrolases named as MutT enzymes. In particular, *Sc*DDP1 shares 33% sequence identity from Val^47^ to Glu^99^ with human MutT-Ap_4_A hydrolase, while human DIPPs reach 47% identity.

Although the Nudix hydrolase superfamily is well characterized structurally, its broad range of catalytic activities and substrate recognition is poorly understood. In particular, the molecular basis for the multiple pyrophosphatase activity on different substrate types, as in the case of *Sc*DDP1, remains unknown. Very recently, while this manuscript was in preparation, the structure of DIPP1 in complex with 1- and 5-InsP_7_ became available (*29*), although no description of catalytic mechanism beyond that already postulated from the InsP_6_ complex (*28*) was provided. Important issues such as InsP_8_ recognition and why *Sc*DDP1 is more active on 1-InsP_7_ over 5-InsP_7_ or InsP_8_ are also still to be addressed. In addition, the human and yeast enzymes, while keeping high homology in the core, present distinctive elements, and not all the InsP-binding residues are conserved among them. The yeast enzymes display an insert (25 residues) not present in the human orthologs, with an unknown function. Last, this family of enzymes, as mentioned, exceptionally presents a pyrophosphatase function on a very diverse set of substrates, such as polyphosphates, Ap*_n_*A, and PP-InsPs, linking essential areas of metabolism within the cell. How *Sc*DDP1 recognizes them and how it is capable of performing its catalytic activity on three completely different substrates are addressed in this current work. Extensive crystallographic studies together with mutagenesis analysis, enzyme assays, use of synthetic substrate analogs (*4*), and qualitative thermal fluorescence shift assays now allow a greater understanding of this family of enzymes.

## RESULTS

### DDP1 is a Nudix enzyme with unique structural traits

**Fig. 1:**
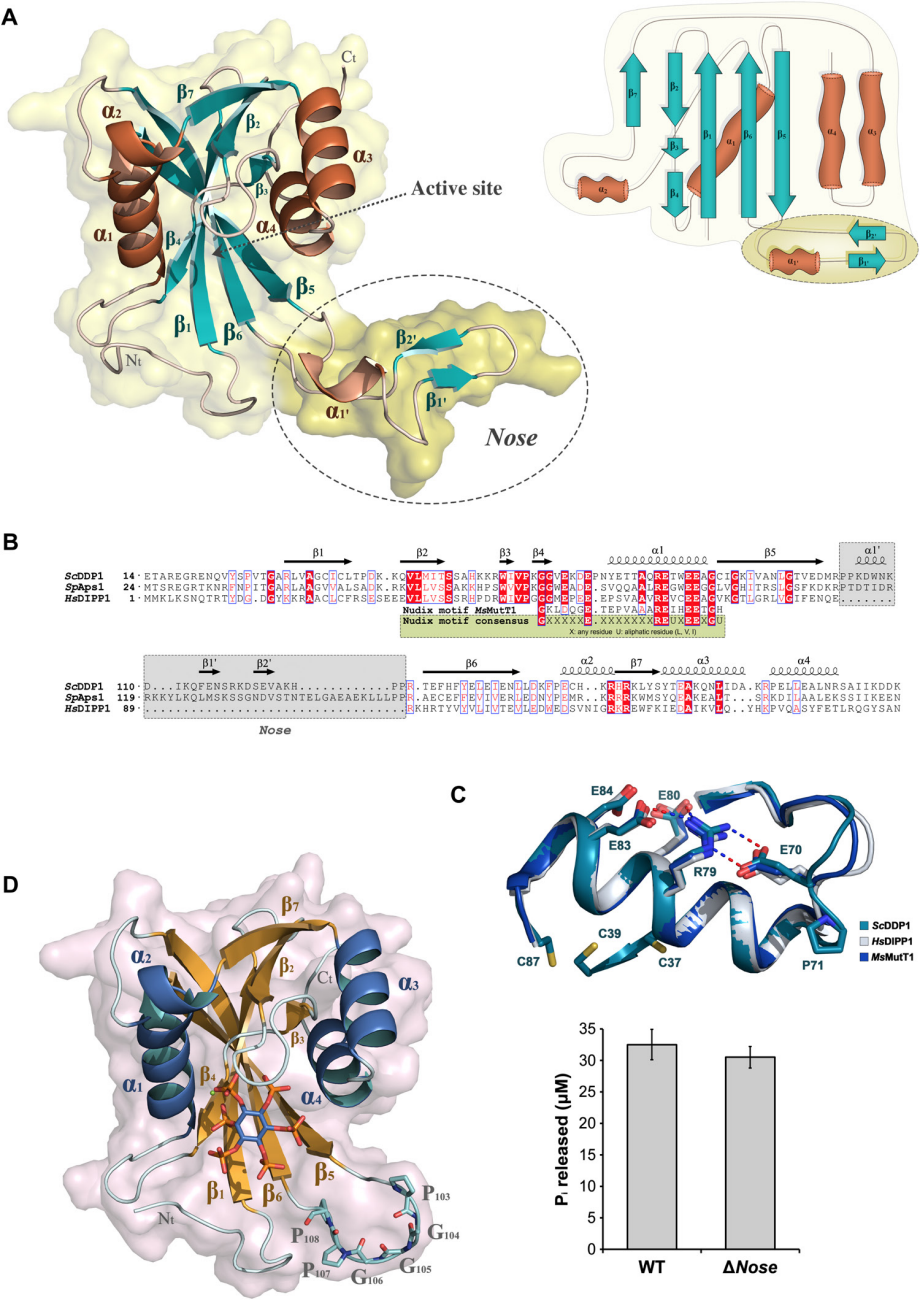
ScDDP1 structure. (**A**) *Sc*DDP1 cartoon representation with transparent surface (left) and topology diagram (right) showing α helices (brown), β strands (teal), and loops (cream). The specific insertion named as “*nose*” is marked as a dashed circle and darker surface. (**B**) Alignment between human and yeast orthologs of DDP1.Top row indicates the secondary structure of *Sc*DDP1. The Nudix consensus and sequence in MutT1, a prototype of Ap_5_A hydrolases, are shown at the bottom. Conserved residues and the *nose* are also highlighted. (**C**) Structural superposition of Nudix motifs in *Sc*DDP1 and its orthologs *Hs*DIPP1 and *Ms*MutT1. (**D**) *Sc*DDP1Δ*nose* cartoon representation highlighting the remaining residues in the nose region as cyan sticks (left) and comparison between *Sc*DDP1 and *Sc*DDP1Δ*nose* relative pyrophosphatase activity at 30 min. The data correspond to triplicates (right). WT, wild type.

*Sc*DDP1, like its human orthologs, adopts the canonical Nudix fold showing two β sheets flanked by helices (Fig. 1A). The active site lies in the crevice situated between both β sheets. The longest helix (α-1) contains the Nudix box (GX_5_EX_7_REUXEEXGU) (Fig. 1B), which displays a β-loop-helix-loop structure that binds the Mg^2+^ ions involved in diphosphate moiety orientation and catalysis. The enzyme MutT1 from *Mycobacterium smegmatis*, a Nudix Ap_4_A hydrolase, can be regarded as a Nudix enzyme prototype (Fig. 1, B and C). The *Sc*DDP1 Nudix region presents some differences from the above motif, including a residue insertion before α1 (Pro^71^) and unique residues at position U (Thr^81^ and Cys^87^) (Fig. 1B). These differences are not found either in its human (DIPPs) or *S. pombe* (Aps1) orthologs (Fig. 1, B and C). Cys^87^ is close to other cysteines (Cys^37^ and Cys^39^); however, the differences mentioned have no impact in the Nudix catalytic region configuration.

The most outstanding feature in the *Sc*DDP1 overall fold is the presence of a remarkable insertion (Pro^103^ to Pro^128^) that projects out of the Nudix scaffold (named as *nose* from now on) (Fig. 1A). This insertion is not present in the human orthologs of *Sc*DDP1; only the *S. pombe* ortholog (Aps1) shows a larger insertion in this region (Fig. 1B). The *nose* has marked flexibility, presenting some degree of disorder in most of our crystal complexes (Table 1). Nevertheless, the electron density map of a few crystals allowed complete tracing, although its atoms presented high B-factors. The *nose* displays an αββ structure that produces crystal contacts with a symmetry-related molecule, which might suggest the putative structure that this region adopts upon wider protein-protein interactions. We have produced a *Sc*DDP1 mutant lacking this region (Δ*nose* mutant), which allowed us to conclude that the *nose* is not essential either for in vitro protein catalytic activity (Fig. 1D) or folding. To assign a function to the *nose*, we investigated whether this motif could play a role in protein oligomerization. An analysis of wild-type (wt)–*Sc*DDP1 crystal contacts with PISA (protein interfaces, surfaces and assemblies) (*31*) reveals that the closest subunits produced a buried area of 946 Å^2^ that represents 10% of the total DDP1 area (9994 Å^2^), suggesting that this may be a crystal packing contact. However, we observed that the *nose* makes intermolecular interactions in a head-to-tail fashion, forming fibers along the crystal, in which proline residues at the edge of the nose act as hinges (fig. S1), raising the question of whether this mode of interaction could arise under some physiological conditions.

**Table 1:** Crystallographic statistics. Values in parenthesis are for the high-resolution shell. *R*_merge_ = ∑_hkl_ ∑_i_ | *I*_i_(hkl) – [*I*(hkl)]| / ∑_hkl_ ∑_i_ *I*_i_(hkl), where *I*_i_(hkl) is the *i*-th measurement of reflection hkl, and [*I*(hkl)] is the weighted mean of all measurements. *R*_pim_ = ∑_hkl_ [1/(*N* - 1)] 1/2 ∑_i_ | *I*_i_(hkl) – [*I*(hkl)]| / ∑_hkl_ ∑_i_ *I*_i_(hkl), where *N* is the redundancy for the hkl reflection. *R*_work_/*R*_free_ = ∑_hkl_ | *F*_o_ – *F*_c_ | / ∑_hkl_ | *F*_o_ |, where *F*_c_ is the calculated, and *F*_o_ is the observed structure factor amplitude of reflection hkl for the working/free (5%) set, respectively. RMS, root mean square.

**Crystal data DDP1**	**InsP_6_**	**5-InsP_7_**	**5-InsP_7_ + Mg**	**PCF**	**PCP-InsP_7_**	**PCP-InsP_8_ + Mg**	**PA-InsP_8_**
Space group	P3_2_21	P3_2_21	P3_2_21	P3_2_21	P3_2_21	P3_2_21	P3_2_21
**Unit cell parameters**							
a, b (Å)	61.78	61.53	61.63	61.81	61.70	61.53	61.67
c (Å)	96.46	95.11	95.98	95.47	95.84	95.50	89.22
**Data collection**							
Beamline	XALOC (ALBA)	XALOC (ALBA)	XALOC (ALBA)	XALOC (ALBA)	XALOC (ALBA)	XALOC (ALBA)	XALOC (ALBA)
Temperature (K)	100	100	100	100	100	100	100
Wavelength (Å)	0.979260	0.979260	0.979260	0.979260	0.979260	0.979260	0.979260
Resolution (Å)	48.23–1.98 (2.03–1.98)	47.55–2.00 (2.05–2.00)	47.99–1.85 (1.89–1.85)	47.74–2.07 (2.13–2.07)	42.92–1.85 (1.89–1.85)	47.75–1.95 (2.00–1.95)	45.83–2.65 (2.78–2.65)
**Data processing**							
Total reflections	266,156 (19,364)	257,514 (19,430)	282,618 (15,331)	250,784 (20,263)	346,358 (20,394)	242,402 (17,739)	108,919 (15,285)
Unique reflections	15,385 (1047)	14,627 (1080)	18,607 (1124)	13380 (1025)	18,625 (1141)	15,810 (1101)	6055 (799)
Multiplicity	17.3 (18.5)	17.6 (18.0)	15.2 (13.6)	18.7 (19.8)	18.6 (17.9)	15.3 (16.1)	18.0 (19.1)
Completeness (%)	100.0 (100.0)	100.0 (100.0)	100.0 (100.0)	100.0 (100.0)	100.0 (100.0)	100.0 (100.0)	99.8 (99.7)
Mean I/σ (I)	32.3 (5.5)	29.1 (4.9)	17.9 (3.2)	23.3 (4.5)	33.2 (6.2)	25.3 (4.3)	25.1 (5.3)
*R*_merge_ (%)	5.0 (66.3)	5.9 (65.3)	7.8 (65.0)	7.3 (67.2)	5.2 (65.2)	5.6 (65.4)	5.8 (65.0)
*R*_pim_ (%)	1.3 (15.9)	1.5 (15.7)	2.1 (18.3)	1.8 (15.6)	1.3 (16.0)	1.5 (16.7)	1.4 (15.2)
Wilson B factor (Å^2^)	36.149	38.954	32.802	40.539	32.470	37.525	80.070
Molecules per ASU	1	1	1	1	1	1	1
**Refinement**							
*R*_work_/*R*_free_ (%)	20.8/22.0	23.4/27.2	25.9/28.1	22.4/23.8	22.3/26.8	21.7/25.6	23.4/27.2
**N°/B average (Å^2^)**							
Protein atoms	1286/49.11	1367/60.70	1367/48.24	1370/70.47	1311/48.39	1319/61.12	1213/114.58
Main ligands atoms	36/74.82	40/92.01	80/82.51	41/133.93	80/82.69	88/74.98	42/120.67
Magnesium atoms	0/0.00	0/0.00	1/63.24	0/0.00	2/65.69	1/45.14	0/0.00
Water molecules	76/49.82	49/58.58	46/44.27	33/55.73	68/49.99	57/47.89	0/0.00
All atoms	1398/49.81	1456/61.49	1498/49.98	1444/71.94	1461/50.36	1465/61.43	1255/114.78
All residues	159	168	168	167	160	163	149
Residues built	20–104, 113–186	20–187	20–187	20–186	20–104, 113–187	20–104, 110–187	21–108, 126–186
**Ramachandran plot**							
Favored (%)	96	97	96	94	96	96	96
Outliers (%)	0	0	0	0	0	0	0
**RMS deviations**							
Bonds (Å)	0.0063	0.0048	0.0047	0.0057	0.0110	0.0049	0.0051
Angles (°)	1.4517	1.2867	1.4137	1.4041	1.8257	1.3154	1.3485
PDB accession codes	7AUI	7AUK	7AUL	7AUM	7AUP	7AUN	7AUO
**Crystal data DDP1**	**polyP15 + Mg**	**Ap_5_ + Ca**	**AMP-PNP**	**K63A**		**E80Q + 1-InsP_7_**	**Δ*Nose* + InsP_6_**
Space group	P3_2_21	P3_2_21	P3_2_21	P3_2_21		P3_2_21	P3_2_21
**Unit cell parameters**							
a, b (Å)	61.67	62.00	61.96	61.78		61.89	62.12
c (Å)	95.73	95.40	95.92	96.18		95.51	92.47
**Data collection**							
Beamline	XALOC (ALBA)	XALOC (ALBA)	XALOC (ALBA)	XALOC (ALBA)		XALOC (ALBA)	XALOC (ALBA)
Temperature (K)	100	100	100	100		100	100
Wavelength (Å)	0.979182	0.979260	0.979300	0.979257		0.979257	0.979257
Resolution (Å)	47.87–1.75 (1.78–1.75)	46.79–2.25 (2.32–2.25)	47.96–1.65 (1.68–1.65)	48.09–1.60 (1.63–1.60)		48.26–2.10 (2.16–2.10)	46.50–2.45 (2.55–2.45)
**Data processing**							
Total reflections	412,767 (23,209)	98,022 (9454)	498,833 (22,396)	395,524 (19,967)		170,940 (14,257)	103,036 (11,250)
Unique reflections	21,864 (1171)	10,184 (945)	26,240 (1299)	28,567 (1393)		13,002 (1051)	7984 (873)
Multiplicity	18.9 (19.8)	9.6 (10.0)	19.0 (17.2)	13.8 (14.3)		13.1 (13.6)	12.9 (12.9)
Completeness (%)	100.0 (100.0)	97.9 (99.5)	99.8 (99.8)	99.7 (99.2)		100.0 (99.9)	99.9 (100.0)
Mean I/σ (I)	34.3 (5.0)	11.7 (3.3)	18.9 (4.8)	28.4 (4.1)		26.1 (4.8)	21.3 (3.2)
*R*_merge_ (%)	4.7 (65.2)	10.5 (65.2)	8.3 (66.6)	5.5 (69.8)		5.3 (55.8)	5.8 (62.4)
*R*_pim_ (%)	1.1 (14.9)	3.5 (21.1)	2.0 (16.4)	1.6 (19.0)		1.5 (15.7)	1.6 (17.8)
Wilson B factor (Å^2^)	33.348	55.090	27.770	17.330		43.232	69.364
Molecules per ASU	1	1	1	1		1	1
**Refinement**							
*R*_work_/*R*_free_ (%)	24.1/27.9	20.5/22.8	20.9/24.2	19.1/21.1		22.5/26.5	20.5/25.4
**N°/B average (Å**^**2**^**)**							
Protein atoms	1367/46.10	1144/67.95	1175/37.20	1369/21.63		1297/63.33	1146/63.28
Main ligands atoms	61/89.84	39/101.40	62/71.74	0/0.00		40/94.98	36/103.45
Magnesium atoms	1/43.12	1 (Ca^2+^)/115.10	2/70.05	1/62.61		1/71.12	1/96.64
Water molecules	81/47.08	37/63.00	112/45.29	178/36.73		38/48.66	15/69.81
All atoms	1510/47.91	1231/68.59	1383/39.50	1549/23.40		1376/63.85	1198/64.60
All residues	167	142	144	167		158	144
Residues built	21–187	21–102, 127–186	21–104, 127–186	21–187		20–103. 113–186	22–165, 169–188
**Ramachandran plot**							
Favored (%)	97	96	98	99		97	97
Outliers (%)	0	0	0	0		0	0
**RMS deviations**							
Bonds (Å)	0.0049	0.0076	0.0074	0.0077		0.0040	0.0040
Angles (°)	1.4204	1.6389	1.5120	1.4439		1.2805	1.3054
PDB accession codes	7AUS	7AUQ	7AUR	7AUT		7AUJ	7AUU

### Promiscuous inositide-binding modes to DDP1 depict a fixed phosphate anchoring pattern

**Fig. 2:**
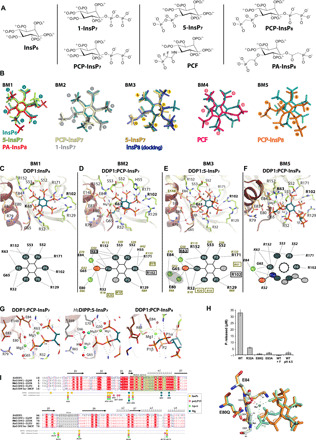
DDP1 inositide recognition. (**A**) Chemical structures of InsPs and their analogues used in this work. (**B**) The five different binding modes (bms) detected for the inositides. InsP_6_ is included in all bms as a reference. (**C**) Structure of *Sc*DDP1:InsP_6_ complex. Protein is shown in cartoons (Nudix region in brown), InsP_6_ in sticks (C, O, and P atoms in teal, red, and orange, respectively), protein residues in lemon sticks (light orange from Nudix region) and hydrogen bonds in dashed lines. (**D**) Structure of *Sc*DDP1:PCP-InsP_7_ complex. Red and green spheres show water and Mg^2+^ ions, respectively. (**E**) Structure of *Sc*DDP1:5-InsP_7_ complex. (**F**) Structure of *Sc*DDP1:PCP-InsP_8_ complex. Bottom panels show the positions for phosphate recognition: six in InsP_6_, PCP-InsP_7_ and 5-InsP_7_, whereas only five in PCP-InsP_8_. Numbering of *Sc*DDP1/*Hs*DIPP1 residues is in black/green respectively. Isoform-specific residues are in squares and Mg2/Mg3 positions are inferred from *Hs*DIPP1:5-InsP_7_ complex. (**G**) Mg^2+^ interactions in *Sc*DDP1:PCP-InsP_7_ (left), *Hs*DIPP1:5-InsP_7_ (middle), and PCP:InsP_8_ (right) complexes. (**H**) (Top) Pyrophosphatase activity (time = 30 min) of wt-*Sc*DDP1, mutants, and wt-*Sc*DDP1 in the presence of Fluor (pH 8) and wt-*Sc*DDP1 (pH 4.5). Data correspond to triplicates. (Bottom) Structural superposition of wt-*Sc*DDP1 (green) and *Sc*DDP1-E80Q (orange) in the presence of 1-InsP_7_. Mg3 is only present in wt-*Sc*DDP1. (**I**) Structural alignment between *Sc*DDP1 and *Hs*DIPP isoforms. The structural secondary elements for *Sc*DDP1 are specified.

We crystallized *Sc*DDP1 in the presence of multiple inositide ligands and synthetic analogs (Fig. 2, Table 1, and fig. S2): the DDP1 product (InsP_6_), DDP1 monopyrophosphorylated substrates (1-InsP_7_ and 5-InsP_7_), nonhydrolyzable analogs of InsP_7_ [PCP-InsP_7_ (1-methylenebisphosphonate analog) and PCF (5-phosphonodifluoroacetamide analog)], and analogs of the bispyrophosphorylated substrate InsP_8_ (PCP-InsP_8_ and the corresponding phosphonoacetate analog, PA-InsP_8_) (Fig. 2A). Because of the high symmetry of InsP_6_ and PP-InsPs, the inositide ring is able to bind in five different modes, named here as binding modes 1, 2, 3, 4, and 5 (Fig. 2B). Only some of these modes are catalytically productive. In all cases, the inositide binds strongly to the active site producing at least 15 polar interactions with the enzyme, mostly with basic residues (Fig. 2, C to F). Our results allowed identification of the primary positions for phosphate recognition, determination of the catalytic positions, and comparison with the human orthologs (Fig. 2).

#### InsP_6_ binding

We have defined InsP_6_ binding as “binding mode 1” (bm1 mode), since its six phosphates make links to the enzyme satisfying all recognition positions, except Pβ of the diphosphate moiety, which is missing in this inositide. In this binding mode, the unique axial phosphate at the 2– position is directed toward the active site (Fig. 2C). All phosphates participate in one to four interactions with protein residues; thus, P1 makes two (Arg^32^), P2 makes three (Arg^32^ bidentate and Gly^65^ main chain), P3 makes four (Arg^152^ bidentate and Ser^53^ side and main chain), P4 makes three (Ser^52^, Lys^63^, Arg^171^), P5 makes three (Arg^102^ bidentate and Arg^129^), and P6 makes one (Arg^129^). Several water-mediated interactions stabilize the inositide. The mentioned residues are involved in the binding of all inositides studied in this work.

#### PCP-InsP_7_ and 1-InsP_7_ binding

PCP-InsP_7_ and 1-InsP_7_ bind into the DDP1 active site in a different way from InsP_6_, which we here define as bm2 mode. We will focus our description on the DDP1:PCP-InsP_7_ complex, since some disorder in the DDP1:InsP_7_ complex structure precluded its full refinement. Here, the axial 2-phosphate projects outside the active site (Fig. 2D). In general, the same *Sc*DDP1 residues that bind InsP_6_ are involved in the PCP-InsP_7_ binding but with an additional interaction with the side chain of His^55^. The 1-PCP analog phosphorus atoms (P1α and P1β) are specifically positioned through interactions with two Mg^2+^ ions [Mg1 and Mg3, as designated previously in work on Nudix enzymes (*32*)] (Fig. 2D). In our DDP1 crystals, the Mg3 ion does not display a full occupancy, and, in consequence, the diphosphate moiety shows two alternate conformations, one bound to Mg1 and Mg3 ions and the other only bound to Mg1 ion (fig. S2). The Nudix residues Glu^84^ and Glu^80^ bind the two Mg^2+^ ions, respectively (Fig. 2G, left). The role of these ions seems to be to fix a proper diphosphate geometry for catalysis. The DDP1-E80Q mutant precludes Mg3 binding, and, in consequence, it selects only the conformation of 1-InsP_7_ with its diphosphate bound solely to Mg1 (see Fig. 2H, bottom). The pattern of interactions for the other phosphates with the enzyme is quite similar, although phosphate positions are exchanged (Fig. 2, B to D). Notably, P2 does not produce any interaction with the enzyme. We can observe a marked conservation of PCP-InsP_7_ binding in DDP1 with 1-InsP_7_ binding in *Hs*DIPP1 (Fig. 2I) (*29*), presenting minor differences due to nonconserved residues: *Hs*DIPP1 presents three specific residues, Arg^10^, Arg^20^, and Arg^41^, for binding P2, P1α, and P4, respectively. Similarly, Lys^63^ and Arg^102^ are *Sc*DDP1-specific residues [Fig. 2, D (bottom) and I].

#### 5-InsP_7_ binding

We obtained several complexes of *Sc*DDP1:5-InsP_7_ in the presence or absence of Mg^2+^. Our results show that 5-InsP_7_ is able to bind in two different modes: bm1 and bm3 (Fig. 2, B and E, and figs. S2 and S3A). We will focus our description on bm3 mode (Fig. 2E), since this is the productive binding mode showing a well-positioned diphosphate moiety. The productive mode is only captured in the presence of magnesium salt but is always mixed with the nonproductive mode in the crystals (fig. S2). Binding of 5-InsP_7_ in bm3 mode is in agreement with the recently published *Hs*DIPP1:5-InsP_7_ complex (*29*). Residues involved in phosphate coordination are the same as identified previously but with subtle differences. In this binding mode, 5-PP (P5α and P5β) is well situated at the catalytic center, similarly to 1-PCP in the *Sc*DDP1:PCP-InsP_7_ complex. We detect a Mg^2+^ ion equivalent to the previously described Mg1. The *Hs*DIPP1:5-InsP_7_ complex displays three Mg^2+^ positions, although with very low occupancy [Fig. 2, E (bottom) and G (middle)] fulfilling the configuration of Mg^2+^ ions in the active site (Mg1, Mg2, and Mg3). The structural alignment between *Sc*DDP1 and *Hs*DIPP1 shows that most Mg^2+^ sites are absolutely conserved [Fig. 2, E (bottom) and I], with the same exceptions described for the PCP-InsP_7_ binding.

Conversely, 5-InsP_7_ in a nonproductive bm1 mode displays the 5-PP moiety opposite to the catalytic center (Fig. 2B and fig. S3A). The fact that 5-InsP_7_, being a substrate for *Sc*DDP1, occupies our *Sc*DDP1 crystals in a nonproductive mode, could be due to the pH of crystallization together with the lack of Mg^2+^ ions in the active site. Our results might also suggest that the catalytically productive mode for 5-InsP_7_ might not be the lowest energetic bound form. In agreement with that idea, it has been reported that *k*_cat_ toward 5-InsP_7_ is five times lower than toward its isomer 1-InsP_7_ (*13*), whereas both compounds display similar Michaelis constant (*K*_m_) values. Therefore, 5-InsP_7_ could fit better in nonproductive binding modes, decreasing the catalysis rate, whereas 1-InsP_7_ tends to fit easily in a productive binding mode.

We also checked the binding of PCF, a 5-InsP_7_ analog, to DDP1. The *Sc*DDP1:PCF complex reveals that PCF binds in a mode named as bm4 (Fig. 2B). This binding mode shares features with bm1, but the diphosphate analog moiety can neither mimic the protein-diphosphate interaction of 5-InsP_7_ in bm1 mode nor effectively chelate a Mg^2+^ ion as 5-InsP_7_ in bm3 mode. In consequence, PCF fits in bm4 mode, and the diphosphate analog moiety remains disordered in the electron density map (fig. S3B).

#### PCP-InsP_8_ binding

We crystallized *Sc*DDP1 in the presence of two nonhydrolyzable InsP_8_ analogs (PCP-InsP_8_ and PA-InsP_8_). PA-InsP_8_, a more distant analog to the more classical PCP approach, binds in bm1 mode, showing a nonproductive form (fig. S3C). PCP-InsP_8_ binds into the DDP1 active site in a different mode from those previously described here (Fig. 2F), from now on named as bm5. The results show that PCP-InsP_8_ keeps P2 at the same position of P2 in the InsP_6_ complex (bm1), but the ring is substantially twisted from this mode, in such a way that only some of the phosphate positions previously described are occupied, while P1α, P6, P5α, and P5β are in new positions (Fig. 2, B and F). From those positions, only P1α presents interactions with the enzyme. The diphosphate 1-PP analog motif is located at the catalytic center, keeping P1β at the same place as P1β in previous productive complexes, while P1α is in a different position. Therefore, the diphosphate analog moiety does not keep an identical position to that found in the InsP_7_ complexes, suggesting that this could be a “pseudo-productive” form. However, Mg1 is found in the structure with a perfect geometry between P1α, P1β, and P2 phosphates (Fig. 2G, right). Nevertheless, we have explored other possible binding modes for InsP_8_ in *Sc*DDP1 by manual docking. To hydrolyze 1-PP and 5-PP bonds, InsP_8_ must bind in two different modes that could be similar to the 1-InsP_7_ and 5-InsP_7_ productive bm2 and bm3 modes, respectively (fig. S4). Clearly, InsP_8_ in bm2 mode with 1-PP located in the Nudix site is not possible, since 5-PP would produce steric clashes with a Ser-loop (52-SSA) and Ile^60^ (fig. S4). However, InsP_8_ in bm3 mode with 5-PP in the catalytic site projects the 1-PP outside only, requiring a little adjusting of Arg^129^ side chain. We propose that bm3 mode, obtained by docking, might be the productive one for InsP_8_ 5-PP hydrolysis, whereas the bm5 mode, obtained experimentally, might be the productive one for 1-PP hydrolysis. This hypothesis can provide an explanation for several experimental findings (*13*): (i) DDP1 hydrolyses InsP_8_ at the 5- position first, which is in agreement with a better situation of the diphosphate moiety to be cleaved in bm3 mode and (ii) produces a very small amount of 5-InsP_7_, which is in agreement with the InsP_8_ “defective binding” in bm5 mode; and (iii) InsP_8_ binding in two different modes could explain a decreased catalytic rate (*13*).

#### Catalytic mechanism for InsPPase activity

Although structures of *Hs*DIPP1 with 1-InsP_7_ and 5-InsP_7_ have been recently reported (*29*), no additional explanations for the catalytic mechanism have been provided beyond those postulated from the *Hs*DIPP1:InsP_6_-Mg-F complex [Protein Data Bank (PDB) 2Q9P] (*28*). As mentioned, *Hs*DIPPs can bind three Mg^2+^ through the Nudix motif, here named as Mg1, Mg2, and Mg3, whereas the *Sc*DDP1:PCP-InsP_7_ complex shows just two of them (Mg1 and Mg3). Our *Sc*DDP1:PCP-InsP_7_ complex was obtained at acidic pH at which the enzyme is not active (Fig. 2H); however, and due to residue conservation, we suggest that fully active *Sc*DDP1 will show three Mg^2+^ ions as do its human orthologs, which in turn were also crystallized at acidic pH. This observation was previously made with other Nudix enzymes (*33*). Whereas Mg1 and Mg3 seem to be essential for substrate binding and phosphate positioning, Mg2 and Mg3 could be necessary for catalytic activity. Mg3 (in cooperation with the missing Mg2) could activate a water molecule to perform nucleophilic attack at Pβ of the diphosphate (Fig. 2G and fig. S4). Glu^80^ and Glu^84^ residues, as well as the main chain of Lys^63^, are holding Mg^2+^ ions, whereas Arg^32^ is particularly close to the diphosphate-cleavable bond, and it could help in intermediate stabilization. We have performed site-directed mutagenesis on Glu^80^, Glu^83^, and Arg^32^. Both mutants on acidic residues produce inactive forms of the enzyme or those with very low activity (Fig. 2H). While Glu^80^ binds Mg3 (its equivalent in *Hs*DIPP1 also binds Mg2), Glu^83^ forms a triad structure with Arg^79^ and Glu^80^, fixing a perfect orientation of these residues. Furthermore, in *Hs*DIPP1, the equivalent residue to the DDP1 Glu^80^ (Glu^66^) is proposed to help in catalytic water molecule coordination (Fig. 2G, middle). We crystallized inactive DDP1-E80Q in complex with 1-InsP_7_, showing also bm2 mode for the inositide; however, there is no Mg3 in the active site as expected, and, in consequence, the diphosphate moiety only fits in a nonproductive conformation (Fig. 2H). Last, the *Sc*DDP1-R32A mutant also presents a very reduced activity, compatible with an important role in diphosphate orientation and/or participation in catalysis (Fig. 2H).

In summary, we have identified multiple sites for phosphate recognition, although there are also secondary positions able to accommodate phosphates. This results in inositide accommodation in several binding modes that are either catalytically productive (bm2, bm3, and likely bm5) or nonproductive (bm1 and bm4). *Sc*DDP1 presents residues (Arg^32^, Lys^63^, Arg^102^, and Arg^129^) showing structural variations across our complexes. Arg^102^ and Arg^129^ of *Sc*DDP1 are located at the beginning of the *nose* (β5-β6 insertion). We propose that these four residues might be important for ligand accommodation and, particularly, for 1-InsP_7_/5-InsP_7_ selection. Similarly, for *Hs*DIPP1, Arg^10^ and Lys^18^ change their conformation notably to bind 1-InsP_7_ or 5-InsP_7_, and the authors suggest their participation in selection of InsP_7_ isomers (*29*). Lys^18^ (*Hs*DIPP1) is equivalent to Arg^32^ in *Sc*DDP1. In contrast, Arg^102^ and Lys^63^ of *Sc*DDP1 are not conserved in *Hs*DIPPs enzymes, pointing to a unique role for them in the yeast kingdom isoforms.

### DDP1 dinucleotide and polyphosphate binding is driven to satisfy the phosphate anchoring sites

DDP1 exerts hydrolytic activities toward very different substrates. Thus, apart from inositide pyrophosphatase activity, it can hydrolyze pyrophosphate bonds in polyphosphate substrates with *n* phosphates (polyP*_n_*) as well as in dinucleotide polyphosphates, particularly Ap_5_A and Ap_6_A. We have crystallized DDP1 in the presence of adenylyl-imidodiphosphate (AMP-PNP), adenosine 5′-triphosphate (ATP), HMP (hexametaphosphate as polyphosphate), and Ap_5_A. All these complexes reveal important information about the multiple functionalities of DDP1.

#### DDP1:Ap_5_A and ATP analogous complexes

**Fig. 3:**
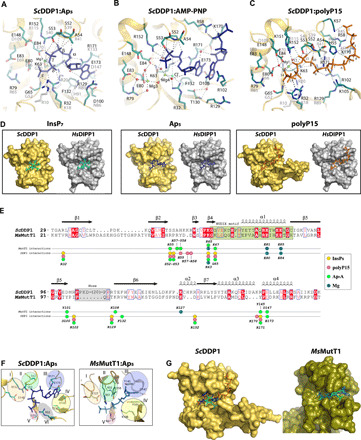
ScDDP1 binding of polyphosphates and dinucleotides. (**A**) Structure of *Sc*DDP1:Ap_5_ complex. *Sc*DDP1 is shown in yellow cartoons, its residues in teal sticks, the Ap_5_ moiety in dark blue, and Mg^2+^ and water as green and red spheres, respectively; residue numbering in *Sc*DDP1/*Hs*DIPP1 in black/gray, respectively; the three specific residues of *Hs*DIPP1 as white sticks; and hydrogen bonds as dashed lines. (**B**) Structure of *Sc*DDP1:AMP-PNP complex. Two interacting molecules of AMP-PNP are shown as blue sticks. (**C**) Structure of *Sc*DDP1:polyP15 complex. PolyP15 is shown as orange sticks. (**D**) Surface representation of *Sc*DDP1 (orange) and *Hs*DIPP1 (gray) showing different ligands: inositide (left, cyan), Ap_5_ (middle, blue), and polyP15 (right, orange). The structures of *Hs*DIPP1 with Ap_5_ and polyP15 have been obtained by structural superposition with *Sc*DDP1 complexes. For clarity, the *nose* is omitted from some panels. (**E**) Structural alignment between *Sc*DDP1 and *Ms*MutT1 enzymes highlighting residues involved in ligands binding. The elements on top represent *Sc*DDP1 secondary structure. (**F**) Distinctive elements in nucleotide binding between *Sc*DDP1 (left) and *Ms*MutT1 (right). (**G**) Surface representation of *Sc*DDP1 (orange) and *Ms*MutT1 (green). The active site delineates a very different cavity to bind PCP-InsP_7_, (cyan) Ap_5_ (blue) or polyP15 (orange) in *Sc*DDP1 and Ap_5_ (blue) in *Ms*MutT1. In *Ms*MutT1, an InsP (cyan) has been placed to show the impossibility of its accommodation.

We obtained complexes of DDP1:Ap_5_A in the presence of Mg^2+^ and Ca^2+^ ions. If no Mg^2+^ is added to the solution, then we observe nonproductive forms. The DDP1:Ap_5_A:Mg^2+^ complex shows more disorder in the dinucleotide position than the DDP1:Ap_5_A:Ca^2+^ complex, but both show essentially the same pyrophosphate-ion-protein coordination and position. We accomplished full structural refinement for the Ca^2+^ complex, which will be described in this section. Only the Ap_5_ portion from Ap_5_A is visible in the electron density map (fig. S2). First, the adenine opens a pocket between the side chains of Arg^171^ and Arg^129^, being sandwiched between both residues and promoting a large shift of Arg^102^ outside this pocket (Fig. 3A and fig. S5). As mentioned before, Arg^102^ and Arg^129^ display a high mobility to accommodate a specific type of substrate. The adenine motif completes its binding through hydrogen bonds between Glu^173^-N6 and Asp^100^-N1 and N6. The ribose moiety is loose in the active site, its furanoside oxygen interacting with a water molecule that links adenine (N3), Pα, Lys^63^, and Arg^171^. Arg^171^ again seems crucial in the Ap_5_ position and conformation. This interaction keeps the nucleotide in a very tight and bent conformation to fit correctly into the active site. On the other hand, the five phosphates (Pα, Pβ, Pγ, Pδ, and Pε) are bound to the same residues involved in inositide recognition. Thus, Pα coordinates to Lys^63^, Arg^171^, and Ser^52^; Pβ coordinates to Ser^52^, Ser^53^, Ala^54^ main chain, and Arg^152^; Pγ coordinates to Ser^53^ and Arg^152^; Pδ coordinates to Arg^32^, Lys^63^, and Mg1; and Pε binds to Arg^32^, Gly^65^ main chain, and Mg1 (Fig. 3A). This structure only shows one Ca^2+^ ion equivalent to the Mg1 previously described and shows similar coordination (Lys^63^ main chain, Pγ, Pδ, Pε, Glu^84^, and a water molecule). This coordination produces a specific geometry to prepare the pyrophosphate motif for attack. The pyrophosphate is in exactly the same position as in both InsP_7_ isomers or analogs in their productive binding modes (bm2 and bm3). The Ap_5_ location and conformation is in agreement with Ap_4_ and AMP being the major hydrolysis products (*15*, *34*). The DDP1:AMP-PNP complex produces the same pattern of interactions for the Pα, Pβ, and Pγ phosphates. Curiously, AMP-PNP shows one Mg^2+^ ion in the Mg3 position and another in a new position coordinated to a Cl^−^ anion (Mg4). For those Nudix enzymes that show ATPase activity (*34*), this could be an inherent scenario for this activity. In this complex, a second nucleotide molecule binds close to the active site, both adenines interacting in an opposite-stacking fashion (Fig. 3B). This second adenine invades the space of Arg^129^, setting it fully apart, and it stacks with Arg^102^ instead. This secondary ATP analog bound in the active site could be a crystallographic artifact; however, its triphosphate moiety resembles some phosphate positions of the polyphosphate chain as we note next. We also obtained ScDDP1:ATP complexes that produced the same information.

#### DDP1:polyP15 complex

We also crystallized DDP1 in the presence of HMP; however, the density we found in the active site is in agreement with a polyphosphate chain of at least 15 units (we checked with the commercial company that this could be a contaminant of the HMP). In this complex (Fig. 3C), apart from the phosphates equivalent to Pα, Pβ, Pγ, Pδ, and Pε positions mentioned before (P10 to P14 here), which conserve the same pattern of interactions described, we see one phosphate after Pε (P15) and 10 phosphates before Pα position (P1 to P9). P13 and P14 (equivalent to Pδ and Pε) conserve interaction with Mg1. The presence of P15 after the cleavable pyrophosphate (P13 to P14) suggests that we observe the substrate ready for the predominant endopolyphosphatase activity described for this enzyme (*11*, *14*). Additional interactions are seen between these phosphates and the enzyme: P1-Lys^57^ and Arg^58^; P3-His^55^, P5-His^55^, Arg^58^, and Lys^170^; P7-His^55^, P8-Arg^102^, and Arg^129^; and P9-Arg^102^. Note that Lys^105^ from the *nose* could participate in polyphosphate binding due to its proximity.

#### DDP1 human orthologs can accommodate polyPs and dinucleotides as ScDDP1

We also tried to understand *Hs*DIPPs activity toward these other kinds of substrates (polyPs and dinucleotides) by comparing our results with the DDP1 structures. Structural superposition between our different *Sc*DDP1 structures and *Hs*DIPP1:InsP_7_ complex shows that the active sites of both enzymes have a broad cavity able to accommodate not only inositides (Fig. 3D, left) but also nucleotides or polyphosphates (Fig. 3D, middle and right). In particular, the superposition of *Sc*DDP1:nucleotide and polyP15 complexes onto *Hs*DIPP1 allowed us to postulate how the human enzyme can recognize these ligands (Figs. 2I and 3D). Apart from the Nudix catalytic residues, most residues involved in the Ap_5_ recognition are conserved, while some of them are specific to *Sc*DDP1 (Lys^63^ side chain) or *Hs*DIPP1 (Arg^10^, Arg^20^, and His^91^; Fig. 3A). Similarly, most residues linking polyP15 are conserved between both enzymes, although each ortholog presents specific residues (*Sc*DDP1: Lys^57^, Lys^63^, and Arg^102^; *Hs*DIPP1: Arg^10^, Arg^20^, and Arg^41^) (Figs. 2I and 3C). These differences can account for different capacities as polyphosphatase and Ap*_n_*A hydrolases.

### Divergences at the phosphate-anchoring sites preclude inositide and polyphosphates binding in MutT1

The structure of *M. smegmatis* MutT1 enzyme (*Ms*MutT1) has been reported in several stages, with Ap_5_ (5XD1), ATP (5XD3), and the product of the reaction (5XD5) revealing important aspects of its possible catalytic mechanism (*32*). A structural comparison between *Sc*DDP1 and *Ms*MutT1 revealed similarities and large differences between both hydrolases (Fig. 3, E and F). We can observe that α1 from the Nudix motif conserves all catalytic residues (Figs. 1, B and C, and 3E). Mg1 and Mg3 positions found in our complexes are conserved with *Ms*MutT1, and we have proposed similar roles for them. Again, *Ms*MutT1 displays a Mg^2+^ ion in an equivalent position to the Mg2 in the *Hs*DIPP1 complex, suggesting that DDP1 should bind this third Mg^2+^ to accomplish its catalytic mechanism. Nevertheless, we can appreciate important differences that explain the ability of DDP1 to also bind inositide and polyphosphate substrates. For simplicity, we have divided the phosphate recognition site into five regions (I, II, III, IV, and V) to display the main differences between both enzymes (Fig. 3F). First, Glu^127^ in *Ms*MutT1 region I helps in Mg2 coordination, while DDP1 Glu^148^, in a similar loop, is relatively close to an activated water in the DDP1:PCP-InsP_7_ complex, suggesting that they are not completely equivalent but could have related roles. The DDP1 residue Arg^152^, essential for either InsPs or polyphosphate coordination, is missing in *Ms*MutT1. Region II seems decisive for substrate selection and conferring different specificity between both enzymes. Residues in this region are essential for phosphate coordination in both enzymes; however, the longer side chains of residues in *Ms*MutT1 delineate a much smaller active site incompatible with the entry of inositides. The shorter side chains in *Hs*MutT1 are compensated by inserted residues having a similar effect in filling this region. Regions III and IV configure the adenine cavity. There is a marked difference between the shape and sequence of loops in this region for both enzymes explaining the different pocket and position for adenine. The stacking found among adenine and aromatic residues would explain a higher affinity in MutT Ap*_n_*A hydrolases. At the end of region IV, and regions V and VI, phosphate binding is covered by different residues in both enzymes (Fig. 3F). While Lys^67^ (*Ms*MutT1) and Arg^32^ (DDP1) play equivalent roles, Lys^108^ in MutT1 binds Ap_5_ phosphates Pα and Pβ positioned at very different place from DDP1 phosphates. In summary, some of the differences highlighted, mainly in region II, explain why DDP1 is active on inositide substrates in addition to polyphosphates and diadenosine phosphates, whereas MutT1 is more restricted to nucleotides and dinucleotide substrates in an extended conformation. The surface representation in Fig. 3G displays a clearly more open active site in *Sc*DDP1, able to accommodate substrates as diverse as InsPs, polyphosphates, and nucleotides. We also suggest that the MutT1 enzyme may not hydrolyze long polyphosphate chains, since residues involved in phosphate binding of the polyphosphate chain in DDP1 (His^55^, Lys^57^, Arg^58^, Arg^102^, Arg^129^, Lys^170^, and Arg^171^) are not conserved in *Ms*MutT1 (Fig. 3E).

### DDP1 stabilization upon substrate binding

**Fig. 4:**
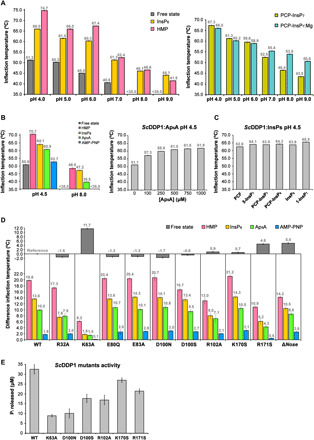
wt-DDP1 and DDP1 mutants analysis. Comparison of inflection temperatures (*T*_i_) obtained by measuring the fluorescence 350/330-nm ratio over a range of temperatures of (**A**). Left: *Sc*DDP1 at different pHs in free state and in HMP and InsP_6_ bound forms. Right: *Sc*DDP1 in the presence PCP-InsP_7_ at different pHs in absence and presence of 5 mM MgCl_2_. (**B**) Left: wt-DDP1 unbound and with the three types of ligands, represented by PCP-InsP_7_ (inositides), Ap_5_A and AMP-PNP (adenine-based), and HMP (polyPs) at pH 4.5 and pH 8.0. Right: Analysis with several concentrations of Ap_5_A (100 μM to 1 mM) at pH 4.5. (**C**) DDP1 in complex with various inositide-based ligands at pH 4.5. (**D**) wt-DDP1 and mutants at pH 4.5 in the absence (top) and presence (bottom) of HMP, InsP_6_, Ap_5_A, and AMP-PNP. Difference inflection temperatures (Δ*T*_i_) have been calculated between each mutant and wild type in absence of ligand as reference (top) or between each sample (either wt or mutant) in the presence of the ligand indicated and its free state (bottom). (**E**) wt-*Sc*DDP1 and *Sc*DDP1 mutants’ pyrophosphatase activity after 30 min. The *T*_i_ values and activity data correspond to triplicates. Error bars show the SDs (not visible when SD ≤ 0.1). When unspecified, ligand concentrations are 100 μM, except for Ap_5_A and AMP-PNP that are 750 μM.

We assessed the thermal stabilization of DDP1 upon binding with all its ligands by recording the 350/330-nm ratio of the fluorescence signal (Fig. 4 and fig. S6). We found that DDP1 is much more resistant to denaturation at acidic pH and, in the presence of its ligands, either with or without Mg^2+^ (Fig. 4A). In agreement, *Sc*DDP1 is prone to crystallize at acidic pH and in the presence of ligands. However, our DDP1 samples at acidic pH are not active (Fig. 2H), in concordance with previous studies that determined optimal DDP1 activity between pH 6.8 and pH 8 (*11*), probably due to the lack of effective Mg^2+^ binding at lower pHs (Fig. 4A, right). Nevertheless, we observed that the relative order of temperatures for denaturation among substrates is kept at acidic and basic pHs (Fig. 4B, left). A direct conclusion from these studies at acidic and basic pH is that the thermal stabilization obtained upon polyphosphates and inositides binding is higher than that achieved by Ap_5_A binding (Fig. 4B, left); in addition, polyphosphate binding produces higher stabilization than inositide binding from pH 4 to pH 8. These findings match with the number of phosphate interactions revealed in the different structures presented in this work, which follow the order: polyPs>PP-InsPs>Ap_5_A. This also agrees with a previous observation that DDP1 degrades polyPs before PP-InsPs when both substrates are present, a fact that the authors suggested could be due to polyPs having more phosphoanhydride bonds than an InsP_7_ (*11*). In addition, the adenine bases in Ap_5_A substrates do not appear to make strong interactions, suggesting that these parts of the ligand do not contribute to a high stabilization beyond the phosphate groups. In agreement, the addition of nearly 10-fold more nucleotide (750 μM) than inositides or polyphosphates (100 μM) is needed to reach complete saturation of DDP1 (Fig. 4B, right). A comparison of thermal stabilization gained upon binding of the different inositide ligands used in this work (Fig. 4C) reveals that all inositides stabilize similarly with no pronounced differences, in agreement with tight binding of all inositides satisfying several phosphate positions.

We also examined the thermal denaturation changes among the different protein mutants produced during this work (R32A, K63A, E80Q, E83A, D100S, D100N, R102A, K170S, R171S, and Δ*nose*) either in the free state or in the presence of inositide InsP_6_, HMP, Ap_5_A, and AMP-PNP (Fig. 4D). We performed S52A/S53A double mutation, but its poor expression as a soluble form and precipitation during purification precluded its analysis. Our analysis provided very interesting results: First, three mutants show prominent increases in thermal stability in the free state (K63A, R171S, and Δ*nose*) as compared to the wild type (Fig. 4D). Both basic residues are very close to each other and other positive charges. Therefore, it seems that removing the positive charges relieves the tension in this highly electropositive active site, especially those for Lys^63^, the mutation of which apparently increased the thermal stability by almost 12°C. As mentioned, Lys^63^ is not conserved in *Hs*DIPPs; however, it is conserved in *Ms*MutT1 enzyme. Although *Sc*DDP1 failed to crystallize in the absence of ligands or analogs, the stabilized *Sc*DDP1-K63A allowed us to solve the structure of DDP1 in the unbound form, revealing that no important structural changes are seen in the active site upon binding beyond minor side chain accommodations of a few basic residues. A retreat from the active site of the mobile Ser-loop (52-SSA) involved in phosphate binding is also observed in this mutant. Similarly, on removing the protein insertion (*nose*) specific to yeast enzymes, the stabilization is notable (5°C), while the protein retains catalytic activity (Fig. 1D). The flexible *nose* has exposed hydrophobic and highly charged residues, the removal of which could result in this stabilization. We must not forget that the loop contains a semi-exposed tryptophan residue that is a major element responsible for the intrinsic fluorescence signal. However, this tryptophan is located outside the folding core, which makes its contribution to denaturation temperature data meaningless.

Next, we compared the stabilization of DDP1 mutants upon binding of different substrates. Comparing InsP_6_ binding among the different DDP1 mutants (Fig. 4D), we observed that mutations involving basic residues (R32A, K63A, R102A, and R171S) display a marked decrease in thermal stabilization upon InsP_6_ binding as compared to the wild type. The larger decrease occurs in K63A and R171S mutants. Note that the four residues bind two or more InsP phosphates (Fig. 2, C to F), which explains these results very well. In the case of polyphosphates, several mutants show less thermal stabilization upon binding than the wild type (Fig. 4D). These differences are notable in K63A, R102A, R171S, and Δ*nose* mutants. This result again is consistent, since all these residues are involved in coordination of the phosphates (Fig. 3C). The smaller DDP1 Δ*nose* mutant stabilization gain upon polyphosphate binding suggests that part of its function could be the recognition of polyphosphates in agreement with our structural findings, which reveal that the *nose* residue Lys^105^ is close to the polyphosphate chain as well as the *nose* border residues Arg^102^ and Arg^129^ (Fig. 3C). Some degree of destabilization upon binding in comparison to wild-type *Sc*DDP1 is observed in the D100S mutant, which might be indirect, since Asp^100^ makes a salt bridge with Arg^102^. Upon Ap_5_A and AMP-PNP binding (Fig. 4D), the higher stabilization lost in relation to the wild type occurred in R171S and K63A. Both residues make an extensive binding with the nucleotides: Lys^63^ binds the Pγ, Pδ, and Pε phosphates from Ap_5_A, contributing to binding and a good geometry in catalysis, whereas the guanidinium group of Arg^171^ stacks with the adenine moiety and coordinates to Pα and Glu^173^, which in turn makes a hydrogen bond with the adenine.

Most of the mutants analyzed retain considerable pyrophosphatase activity in relation to the wild type (Fig. 4E). Some kind of activity decrease is in concordance with a less efficient polyphosphate binding being more evident in the case of K63A mutant (Fig. 4E). However, three of the mutants analyzed exhibit a marked decrease in activity, as seen before (Fig. 2H). E80Q and E83A impede the metal coordination and, in consequence the catalysis, but they have no effect in substrate binding as seen in this section. In contrast, changes in Arg^32^ produce a moderate decrease in most substrates binding being marked on inositide substrates (Fig. 4D). Notably, the R32A mutant shows a marked decrease of activity (Fig. 2H), suggesting that Arg^32^ participates in substrate binding but mainly in diphosphate positioning and catalysis, probably by neutralizing the negative charge developed in the transition state.

In summary, residues Arg^32^, Lys^63^, and Arg^171^ are essential for phosphate binding in all the three kinds of ligands (inositide, dinucleotide phosphates, and polyphosphates). Notably, Arg^102^ is critical for inositide and polyphosphate binding, being less critical for dinucleotide binding. This agrees with Arg^102^ being displaced upon adenine entrance (fig. S5). Last, the *nose* seems to have a marked effect in polyphosphate binding, although its effect on inositide and Ap_5_A binding is also evident. Some of the crystallographic structures obtained in this work show affinity sites for InsP molecules outside the active site where the *nose* participates, suggesting also that the *nose* might have a role in substrate recruitment.

## DISCUSSION

In the present work, we have provided the crystal structure of DDP1 from *S. cerevisiae* with a wide collection of possible substrates and analogs. The fascinating ability of DDP1 to bind very dissimilar substrates can be rationalized from this valuable structural information. DDP1 displays a ligand recognition ability absolutely dominated by phosphate moieties in all substrates. Thus, the phosphates of polyphosphate and diadenosine substrates delineate, through bond torsions, a pathway fully compatible with that for the positions of fully phosphorylated inositides, emulating a similar phosphate pattern substrate recognition. However, the higher stabilization achieved by inositide and polyphosphate substrates suggests an optimal fit for these kinds of substrates.

Our results not only reveal structural similarities of *Sc*DDP1 with human orthologs (*Hs*DIPP1) and Nudix MutT hydrolases but also provide essential differences that can explain their different features and functionalities. *Sc*DDP1 shows a marked extension projecting out from the Nudix core (that we name *nose*), which seems to be specific for this family in the yeast kingdom. We have designed a *nose*-lacking DDP1 mutant producing an active and folded protein that depicted the outstanding influence of the *nose* not only in polyphosphate binding but also in all kinds of substrates. Although direct interactions of *nose* with the ligands have not been observed, the presence of secondary inositide molecules close to the *nose* suggests a possible role of this region in substrate recruitment. Whether the nose has additional role in protein oligomerization, in signaling through interaction with other proteins, or in activity regulation, for example, by closing the entrance to the active site, awaits further investigations. As expected, the essential Nudix catalytic residues are well conserved among all these enzymes. Many of the phosphate-coordinating residues are shared between *Sc*DDP1 and its human orthologs, while only a few are shared with the MutT-related enzymes. However, *Sc*DDP1 has three residues involved in substrate coordination, two specific (Lys^63^ and Arg^102^) and one conserved with *Hs*DDP1 (Arg^129^), which are involved in different inositide substrate accommodation and selection through movement of their long and flexible side chain. Moreover, the two latter residues (Arg^102^ and Arg^129^) exhibit large conformational changes that configure a very different cavity for nucleotide recognition.

Focusing on inositide binding, we found seven positions for phosphate recognition: Six positions are defined by the InsP_6_ complex plus a seventh defined by the “Pβ-to-be-cleaved” position in the PCP-InsP_7_ complex (Fig. 2, C and D). As InsP_6_ and further phosphorylated InsPs present high symmetry, we found a variety of nonproductive and productive binding forms that fit nicely into the DDP1 active site, satisfying in most cases six of the above-mentioned phosphate recognition positions. InsPs and analogs can fulfill this binding pattern in five binding modes, although exceptionally in the case of PCP-InsP_8_ (bm5), just four phosphate positions are satisfied. Note that even enzyme substrates such as 5-InsP_7_ can bind in nonproductive forms. We think that an explanation for obtaining nonproductive forms could be that our complexes were captured at an acidic pH in which the enzyme is not active, which in turn can account for the suboptimal occupancy of Mg^2+^ ions found in the active site. However, we find a correlation between the binding modes and the kinetic data reported by Kilari *et al*. (*13*). Thus, while the *K*_m_ data for 1-InsP_7_, 5-InsP_7_, and InsP_8_ are quite similar, the *k*_cat_ is significantly greater for 1-InsP_7_ than for the two other substrates. It has been proposed that a faster catalysis on 1-InsP_7_ can compensate for its lower levels in cells (*13*). Among our *Sc*DDP1-inositide complexes, while PCP-InsP_7_ and 1-InsP_7_ tend to bind in a productive form, 5-InsP_7_ binds preferentially in a nonproductive form. This fact could explain the differences in *k*_cat_ between both InsP_7_ isomers while keeping similar *K*_m_ values. A quite similar explanation could be suggested for InsP_8_. *Sc*DDP1 must bind InsP_8_ in two different ways to hydrolyze the PP moieties at both 1- and 5 positions. Published data conclude that DDP1 preferentially hydrolyzes the diphosphate at the 5-PP position in InsP_8_ (*13*). In the present work, we solved the structure of a *Sc*DDP1:PCP-InsP_8_ complex in a pseudo-productive form for 1-PP hydrolysis, which neither satisfies the six phosphate positions for recognition nor positions the diphosphate moiety exactly as in other productive complexes, although it is still coordinated with the Nudix recognition motif through a Mg^2+^ ion. Thus, we propose here a model for InsP_8_ binding based on 5-InsP_7_ that satisfies both phosphate-binding positions and 5-PP diphosphate fitting. Therefore, we suggest that InsP_8_ binds in a perfectly productive form to hydrolyze 5-PP, while it acquires a pseudo-productive form to hydrolyze 1-PP. This could explain why hydrolysis at the 1-PP position is less favored than hydrolysis at the 5-PP position and the lower activity rate toward InsP_8_ than 1-InsP_7_, since InsP_8_ binding could result in two alternate ligand positions, one of them being less efficient.

The *Sc*DDP1 structure also reveals which particular region is responsible for inositide accommodation, in contrast to Ap*_n_*A hydrolases, such as MutT enzymes that cannot act on these substrates. The *Sc*DDP1 region 52-SSA presents two serine residues that are directly involved in phosphate coordination in all kinds of substrates recognized by the enzyme. This region always presents some degree of disorder in our complexes, and the double mutant on serine residues (S52A/S53A) produces a nonstable form of DDP1. Therefore, we conclude that these serine residues are essential for protein folding and/or stability and for binding all substrates, and they are responsible for the ability of *Sc*DDP1 to accommodate inositides. Equivalent regions in Ap*_n_*A hydrolases from the Nudix family show residues with long side chains (Fig. 3, E and F) or longer loops, and while they are also able to coordinate phosphates, they fill the active site space destined to accommodate the inositide ligands in *Sc*DDP1. In consequence, in DDP1, Ap_5_A- and polyphosphate-based substrates adopt conformations that follow the contour of the inositol phosphates instead of acquiring an extended conformation as in MutT enzymes. This tightened and constrained conformation of the polyphosphate chain in the *Sc*DDP1 active site also explains why DDP1 is an Ap_6_A and Ap_5_A hydrolase and not an Ap_4_A hydrolase as well as the main hydrolysis products obtained in each case (*15*).

Our study also shows how a longer polyphosphate chain binds in a Nudix enzyme. Following the serines mentioned above, the 54-AHKKR motif is involved in polyphosphate binding beyond the defined positions for phosphate recognition. This motif is not absolutely conserved in *Hs*DIPP1 enzymes or in Ap*_n_*A hydrolases; therefore, we suggest that this could select different polyphosphate chain substrates or account for a possibly better activity of DDP1 on polyphosphate substrates (*11*). However, an additional contribution by the *nose* cannot be discarded. In addition, our structure of polyP binding in the *Sc*DDP1 active site explains the endopolyphosphatase activity determined for this enzyme (*11*), although it does not exclude the possibility of an exopolyphosphatase activity.

In conclusion, the structural knowledge provided by our *Sc*DDP1 complexes generates a vast amount of information most valuable for several fields. First, many efforts are being undertaken to understand the metabolism of PP-InsPs due to the relevance of these compounds in essential cell functions such as apoptosis (*9*) and other emerging roles (*10*). Therefore, our DDP1 complexes add invaluable data to this inositide field. Second, Nudix enzymes represent a well-characterized superfamily able to act on very different substrates. To our knowledge, this is the first structural study showing how a Nudix enzyme shares three kinds of substrates including inositides, and the structural basis for this uniqueness has been established. But understanding the structure and function of multiple substrate enzymes is also a challenge that goes beyond enzymology or structural biology; how an enzyme attains specificity for its substrates and maintains the ability to act on different substrates represents a great challenge from both an evolutionary and a structural point of view. In addition, we depict the structural basis for recognition of a complete set of substrates and analogs, providing a huge source of information to design structure-based inhibitors for these enzymes. *Sc*DDP1, as well as *Hs*DIPP1, contribute to the regulation of inositide, polyphosphate, and dinucleotide metabolism and considering the participation of such molecules in signaling and other events, it follows that manipulating enzyme activity could potentially have important effects in health. It is now established that fungal infections and their virulence are related to phosphorus uptake. On the one hand, mobilization of phosphate from polyphosphates is one of the first responses evoked in fungi to phosphate limiting conditions and on the other InsP_7_, a signaling molecule for the PHO pathway, participates directly in phosphate sensing and signaling through binding to SPX domains (*25*). Moreover, it is known that InsP_7_ is required for *Cryptococcus neoformans* pathogenicity (*35*, *36*) and that *Sc*DDP1 is implicated in phosphate homeostasis of *S. cerevisiae* by degrading InsP_7_ and polyphosphates. Our findings presented here provide a tool to approach improved treatments for fungal infections. We propose that Lys^63^ and Arg^102^ are unique residues in yeast orthologs that should be considered for specific inhibitor design.

## MATERIALS AND METHODS

### PP-InsPs synthesis

PCP-InsP_8_ or 1,5-[PCP]_2_-InsP_4_ (*37*), PA-InsP_8_ or 1,5-[PA]_2_-InsP_4_ (*37*), and PCF or 5-PCF_2_Am-InsP_5_ (*38*) were synthesized as previously described. PCP-InsP_7_ or 1-PCP-InsP_5_ was synthesized as previously described for 1,5-[PCP]_2_-InsP_4_ (*37*) but starting from 1d-4*-O*-benzyl-2,3:5,6-di-*O*-isopropylidene *myo*-inositol. The PP-InsPs 1-InsP_7_ and 5-InsP_7_ were synthesized using similar methods to those previously described (*39*, *40*). All the above synthetic compounds were purified by ion-exchange chromatography and fully characterized by ^1^H, ^31^P, and ^13^C nuclear magnetic resonance spectroscopy.

### Cloning and site-directed mutagenesis

The *DDP1* gene was obtained from *S. cerevisiae* genomic DNA. For its bacterial recombinant expression, *Scddp1* cDNA was inserted into sites Bam HI/Not I of pKLSL_t_ vector, which expresses the target protein in fusion with LSL_t_ (a fragment of *Laetiporus sulphureus* lectin) (*41*). The cDNA was amplified by polymerase chain reaction using the primers 1 and 2 (table S1) and inserted into the vector by megaprimer-mediated molecular cloning (*42*). Point and double DDP1 mutants were obtained by site-directed mutagenesis using as template *Sc*DDP1/pKLSL_t_, and the primers were indicated in table S1. To produce a truncated DDP1 enzyme lacking the Pro^104^-His^126^ residues (DDP1Δ104–126 or DDP1Δ*nose*), we designed the primers 23 and 24 to change fragment 104–126 for three Gly residues by site-directed mutagenesis using the *Sc*DDP1/pKLSL_t_ plasmid as a template.

### Protein expression and purification

wt-DDP1 fused to LSL_t_ protein (LSL_t_-DDP1) was expressed in *Escherichia coli* Rosetta2(DE3) (Novagen) strain after its transformation with the plasmid *Sc*DDP1/pKLSL_t_. Bacteria were grown in LB medium supplemented with kanamycin and chloramphenicol (both 50 μg/ml) at 289 K overnight (16 hours) and at 303 K until an OD_600_ (optical density of 600) of 0.7 to 0.8 was reached. Expression was induced with 0.4 mM isopropyl-β-d-thiogalactopyranoside for 5 or 6 hours at 303 K. Cells were harvested by centrifugation, and pellets were stored at 193 K until use. DDP1 mutants were expressed exactly as wt-DDP1. Pellets were resuspended in buffer A [20 mM tris-HCl (pH 8.0), 150 mM NaCl, and 1 mM dithiothreitol (DTT)] plus deoxyribonuclease (4 μg/ml; Sigma-Aldrich). After sonication, the filtrated lysate was applied onto a Sepharose CL-6B column (GE Healthcare) equilibrated in buffer A. Protein was washed with buffer A and eluted by adding 0.2 M lactose to the buffer. The fusion protein LSL_t_-DDP1 was diluted threefold with 20 mM tris-HCl (pH 8.0), loaded onto a heparin column (HiTrap Heparin, GE Healthcare), washed with buffer B [20 mM tris-HCl (pH 8.0), 50 mM NaCl, and 1 mM DTT], and eluted with a NaCl 1 M gradient. The fused protein was cleaved overnight with tobacco etch virus protease (protease-protein mass ratio of 1:40) with slow rolling at 277 K. A second Sepharose column was used to separate wt-DDP1 from LSL_t_ and the remaining fusion protein. wt-DDP1 was further purified by size-exclusion chromatography using a Superdex 75 10/300 GL column (GE Healthcare) equilibrated in buffer A. Last, wt-DDP1 was concentrated and stored at 193 K. We obtained 5 mg of pure wt-DDP1 per liter of bacteria culture at concentration of 6.25 mg/ml. The purity of all the samples was confirmed by SDS–polyacrylamide gel electrophoresis. DDP1 mutants were purified with the same protocol as wt-DDP1.

### DDP1 cocrystallization with ligands

wt-DDP1 was cocrystallized by the sitting drop vapor diffusion method at 291 K, with three groups of ligands: based on inositol, polyphosphates, or adenosine. The initial crystallization conditions [20% (v/v) polyethylene glycol, molecular weight 6000, 0.1 M sodium acetate (pH 5.0), and lithium chloride] were found after setting up four commercial crystallization screenings: PACT (Jena Bioscience), Crystal Screen (Hampton Research), Index (Hampton Research), and JCSG (Jena Bioscience) using a Nanodrop robot (Innovadyne Technologies Inc.), 250:250 nl protein:precipitant drop ratio in 96-well sitting drop plates. PCT Pre-Crystallization Test (Hampton Research) was performed to optimize protein concentration at 6 mg/ml.

All wt-DDP1 crystals were obtained only in the presence of any of the three groups of ligands. The best cocrystallization conditions for each ligand were discovered by optimization in 48-well plates (Hampton Research). Drop sizes and protein:precipitant ratio were adjusted for each case. Final crystallization conditions for each crystal complex are listed in table S2. Sequentially microseeding and a protein:precipitant 3:1 ratio were necessary to improve the quality of the *Sc*DDP1Δ*nose* crystals. Several cryoprotectants were tested, being usually the original crystallization conditions with slightly increased precipitant concentration and plus 25 to 30% (v/v) of cryoprotectant. After gradually changing the crystal drop solution by cryoprotectant solution, crystals were flash cooled in liquid nitrogen (77 K). We performed similar crystallization experiments to obtain structures for mutants and, in the case of DDP1-K63A mutant, obtained crystals in the absence of any ligand.

### Structural determination

Diffraction data were collected at 100 K in beamline BL13-XALOC, in ALBA Synchrotron (Barcelona, Spain) using a PILATUS 6M detector (*43*). Each dataset was indexed, integrated, and scaled in the XDS program (*44*), while merging, molecular replacement and refinement were carried out with the CCP4 package: Aimless (*45*), MOLREP (*46*), REFMAC5 (*47*), and Coot (*48*). All DDP1 crystals share the same spatial group (P3_2_21) and similar unit cell parameters with one molecule in the asymmetric unit. First, the human DIPP1 structure (PDB code 2FVV) was used as a model for molecular replacement of DDP1:InsP_6_ structure. Our refined models became the template for the rest of the datasets. Diffraction statistics and final refinement parameters are shown in Table 1. Protein rendering was performed with PyMOL (*49*) and structural alignments with ESPript and SeaView programs (*50*, *51*).

### Activity assays

Activity assays were performed using the Malachite Green Phosphate Assay Kit (Sigma-Aldrich). In this assay, free orthophosphate generated in the sample reacts with molybdate and green malachite creating a colorimetric complex, measurable in the visible spectrum with high sensitivity. Activity of 10 nM wt-DDP1 was quantified with substrates HMP (5 to 50 μM) at 303 K, taking an aliquot every 2 to 3 min for 30 min. Reactions were carried out in buffer C [100 mM tris-HCl (pH 7.2), 150 mM NaCl, 1 mM DTT, and 2 mM MgCl_2_] (*11*). Transparent 96-well plates (Corning Costar) and a FLUOstar Omega plate reader (BMG LABTECH) were used to measure absorbance at 620 nm of each sample. The activities of mutants were also quantified using the same procedure as for wt-DDP1. All data presented correspond to the mean of triplicate measurements.

### Fluorescence thermal shift assays

Protein denaturation was recorded by measuring intrinsic protein fluorescence at 330 and 350 nm over a range of temperatures using Tycho NT.6 equipment (NanoTemper Technologies). The changes in fluorescence signal indicate transitions in the folding state of a protein. The temperature at which a transition occurs is called the inflection temperature (*T*_i_). The fluorescence recorded during the thermal run is plotted as 350/330-nm ratio and used to calculate *T*_i_ automatically by the Tycho NT.6 software. The protocol optimized by the manufacturer consists of 3 min of heating, 10 μl of sample in a very thin capillary, and a ramp from 35° (308 K) to 95°C (368 K). For the experiment, the DDP1 concentration was diluted to 0.25 mg/ml (11.5 μM) with buffer A and pH adjusted with final buffer of 0.1 M [MMT (pH 4.0), sodium acetate (pH 4.5), sodium acetate (pH 5.0), bis-tris (pH 6.0), Hepes (pH 7.0), tris-HCl (pH 8.0), or MMT (pH 9.0) depending on each experiment]. It was then mixed with ligands between 100 and 1000 μM.

## SUPPLEMENTARY MATERIALS

Supplementary material for this article is available at http://advances.sciencemag.org/cgi/content/full/7/17/eabf6744/DC1

## Appendix

View/request a protocol for this paper from *Bio-protocol*.
